# Unveiling spontaneous renal tubule-like structures from human adult renal progenitor cell spheroids derived from urine

**DOI:** 10.1093/stcltm/szaf002

**Published:** 2025-03-29

**Authors:** Francesca Giannuzzi, Angela Picerno, Silvia Maiullari, Francesca Montenegro, Antonella Cicirelli, Alessandra Stasi, Giuseppe De Palma, Vito Francesco Di Lorenzo, Giovanni Battista Pertosa, Paola Pontrelli, Michele Rossini, Nunzia Gallo, Luca Salvatore, Vincenzo Di Leo, Mariella Errede, Roberto Tamma, Domenico Ribatti, Loreto Gesualdo, Fabio Sallustio

**Affiliations:** Department of Interdisciplinary Medicine, University of Bari “Aldo Moro,” 70124 Bari, Italy; Department of Interdisciplinary Medicine, University of Bari “Aldo Moro,” 70124 Bari, Italy; Department of Interdisciplinary Medicine, University of Bari “Aldo Moro,” 70124 Bari, Italy; Department of Interdisciplinary Medicine, University of Bari “Aldo Moro,” 70124 Bari, Italy; Department of Interdisciplinary Medicine, University of Bari “Aldo Moro,” 70124 Bari, Italy; Department of Precision and Regenerative Medicine and Ionian Area, University of Bari “Aldo Moro,” 70124 Bari, Italy; Institutional BioBank, Experimental Oncology and Biobank Management Unit, IRCCS Istituto Tumori “Giovanni Paolo II,” 70124 Bari, Italia; Urology Unit, IRCCS Istituto Tumori “Giovanni Paolo II,” 70124 Bari, Italia; Department of Precision and Regenerative Medicine and Ionian Area, University of Bari “Aldo Moro,” 70124 Bari, Italy; Department of Precision and Regenerative Medicine and Ionian Area, University of Bari “Aldo Moro,” 70124 Bari, Italy; Department of Precision and Regenerative Medicine and Ionian Area, University of Bari “Aldo Moro,” 70124 Bari, Italy; Department of Engineering for Innovation, University of Salento, 73100 Lecce, Italy; Typeone Biomaterials Srl, 73021 Calimera, Lecce, Italy; Department of Engineering for Innovation, University of Salento, 73100 Lecce, Italy; Typeone Biomaterials Srl, 73021 Calimera, Lecce, Italy; Department of Precision and Regenerative Medicine and Ionian Area, University of Bari “Aldo Moro,” 70124 Bari, Italy; Department of Translational Biomedicine and Neuroscience “DiBraiN,” University of Bari “Aldo Moro,” 70124 Bari, Italy; Department of Translational Biomedicine and Neuroscience “DiBraiN,” University of Bari “Aldo Moro,” 70124 Bari, Italy; Department of Translational Biomedicine and Neuroscience “DiBraiN,” University of Bari “Aldo Moro,” 70124 Bari, Italy; Department of Precision and Regenerative Medicine and Ionian Area, University of Bari “Aldo Moro,” 70124 Bari, Italy; Department of Precision and Regenerative Medicine and Ionian Area, University of Bari “Aldo Moro,” 70124 Bari, Italy

**Keywords:** renal progenitors, spheroids, tubule-like structures, IgA nephropathy, regenerative medicine

## Abstract

The rapidly developing field of renal spheroids and organoids has emerged as a valuable tool for modeling nephrotoxicity, kidney disorders, and kidney development. However, existing studies have relied on intricate and sophisticated differentiation protocols to generate organoids and tubuloids, necessitating the external administration of multiple growth factors within precise timeframes.

In our study, we demonstrated that human adult renal progenitor cells (ARPCs) isolated from the urine of both healthy subjects and patients can form spheroids that naturally generated very long tubule-like structures. Importantly, the generation of these tubule-like structures is driven solely by ARPCs, without the need for the external use of chemokines or growth factors to artificially induce this process. These tubule-like structures exhibit the expression of structural and functional renal tubule markers and bear, in some cases, striking structural similarities to various nephron regions, including the distal convoluted tubule, the loop of Henle, and proximal convoluted tubules. Furthermore, ARPC spheroids express markers typical of pluripotent cells, such as stage-specific embryonic antigen 4 (SSEA4), secrete elevated levels of renin, and exhibit angiogenic properties. Notably, ARPCs isolated from the urine of patients with IgA nephropathy form spheroids capable of recapitulating the characteristic IgA1 deposition observed in this disease. These findings represent significant advancements in the field, opening up new avenues for regenerative medicine in the study of kidney development, mechanisms underlying renal disorders, and the development of regenerative therapies for kidney-related ailments.

## Graphical Abstract

**Graphical Abstract:**
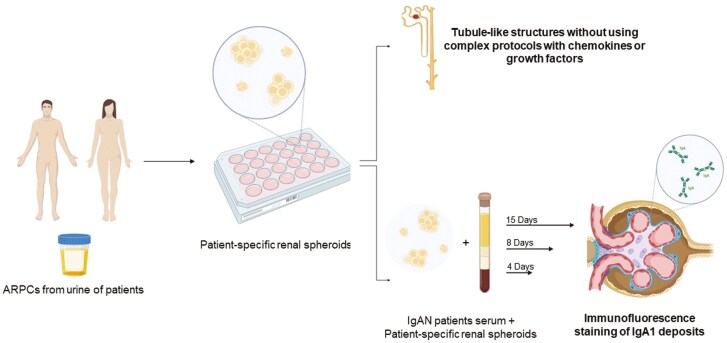

**Significance statement**

Renal progenitors isolated from the urine of patients can form spheroids expressing stem cell markers and can successfully expand. When generated by a mixed cell population of adult renal progenitor cells and CD133^−^ cells, these spheroids can naturally form very long tubule-like structures without using complex protocols with chemokines or growth factors. This is a significant advancement in the field, especially considering that these cells can be isolated from the urine of patients to generate spheroids capable of recapitulating diseases, as we showed for IgA Nephropathy.

## Introduction

Chronic kidney disease (CKD) affects up to 10% of the world’s population and poses a significant global health challenge with substantial financial implications for healthcare systems. The progression of CKD is expedited by repeated occurrences of acute kidney injury.^1,2^ This condition is characterized by tubular atrophy and interstitial fibrosis.^3,4^ Under normal physiological conditions, intrinsic repair mechanisms facilitate the preservation of normal kidney parenchyma; however, failure to repair can occur in cases of severe and/or repetitive injury.

Nevertheless, renal tubular cells can repair damaged renal tissue.^5-7^ Injured proximal tubules (PTs) are also repaired by human adult renal progenitor cells (ARPCs), which exhibit various regenerative properties, including differentiation into renal proximal tubular epithelial cells or podocytes,^8-12^ secretion of reparative factors,^13,14^ and immunomodulation.^15,16^

Recent advances in stem cell biology have allowed the creation of spheroids and kidney organoids from human and mouse pluripotent stem cells (PSCs) and renal stem cells.^17-22^

Spheroids are 3D in vitro microscale tissue analogs that mimic crucial physiological aspects observed in vivo. These include the deposition of extracellular matrix, complex multicellular architecture, and challenges related to mass transfer.

Complex cell-to-cell adhesion and cell-to-matrix interactions in these cell aggregates lead to the formation of gradients for nutrients, gases, growth hormones, and signaling factors. The microenvironment of cells found in tissues is replicated by these structures. Compared with 2D cultures, spheroids serve as suitable in vitro models for studying drug penetration and provide superior systems for drug testing.^23-26^ Spheroids can transform into organoids consisting of multisegmented nephron epithelial cells.^19,27^ The rapidly developing field of renal organoids has proven useful in simulating nephrotoxicity, kidney disorders, and kidney development.^17,28-31^ Organoids have been successfully generated from induced PSCs and embryonic stem cells, as well as from human adult kidney tissue and urine.^22,29,32-34^ However, in all these studies, the generation of organoids and tubuloids necessitated the use of sophisticated and complex differentiation protocols involving the external administration of various growth factors and adherence to precise and specific timescales.^30,35-39^ Recently, culture protocols have been developed to generate “iPSC organoid-derived tubuloids” from differentiated iPSC kidney organoids.^22,40-42^ However, challenges such as decreased expansion ability, fibrosis accumulation, and cyst development after prolonged culture (beyond day d7 + 18, depending on the procedure) pose obstacles for various applications.^40-43^

Here, we demonstrate that human ARPCs isolated from the urine of both healthy subjects and patients can form spheroids, exhibiting renal progenitor properties and recapitulating aspects of organoids. Importantly, these spheroids naturally develop into elongated tubule-like structures without the need for the external administration of chemokines or growth factors to induce the process artificially. This stands in contrast to the current methodologies employed for generating renal organoids and tubuloids, which typically rely on such external factors for induction. Tubule-like formations exhibited structural and functional markers of renal tubules, including CD249, aminopepidase N, ZO-1, uromodulin, and Lotus tetragonolobus lectin (LTL). Additionally, in some cases, they share many structural similarities with some regions of nephrons, such as the distal convoluted tubule, the loop of Henle, and the proximal convoluted tubules. Moreover, our study revealed that ARPCs isolated from patients with IgA nephropathy formed spheroids capable of recapitulating the hallmark IgA1 deposition characteristic of the disease.

## Methods

### Cell culture

Human CD133^+^/CD24^+^ ARPCs were isolated from human urine samples of healthy donors aged 22-60 years^44,45^ as well as from portions of the normal cortex of patients who underwent radical or partial nephrectomy for renal clear-cell carcinoma and were characterized as previously described.^13-16,46,47^ All participants provided written consent, and all experimental procedures were carried out according to the principles of the Declaration of Helsinki and were approved by our institutional ethics review board (Independent Ethical Committee of Policlinic Hospital of Bari). Fresh urine from each donor was centrifuged for 10 minutes at 1800 rpm. The supernatant was discarded, and the sediment was washed with 1 × phosphate-buffered saline (PBS). The cells were suspended in fresh endothelial cell growth medium (EGM) (Lonza, Basel, Switzerland) supplemented with 20% fetal bovine serum (FBS) (Corning, New York, USA), 1% penicillin-streptomycin (P/S) (Euroclone, Milan, Italy) and 1% amfotericin (Euroclone, Milan, Italy) at 37 °C and 5% CO2. Isolation of renal fractions was performed as reported elsewhere^14,48^; both glomerular and tubular fractions were recovered, and tARPCs and gARPCs were maintained in EGM supplemented with 20% FBS at 37 °C and 5% CO2. Human renal proximal tubule epithelial cells (RPTECs) were purchased from ATCC-LGC (ATCC-LGC Standards S.r.l., Sesto San Giovanni, Milan, Italy) and Lonza (Lonza, Basel, Switzerland). Cells were maintained in Dulbecco’s minimal essential medium/Ham’s F12 (DMEM/F12) supplemented with 20% FBS, 1% L-glutamine, 1% P/S at 37 °C, and 5% CO2.

### Spheroid generation

To generate and culture renal spheroids, we used a cell population composed of ARPCs isolated from urine or nephrectomy and CD133^−^ cells from the same patient. Among the mixed cell population, ARPCs accounted for 15%-50% of the total cells. The cells were then plated in the microspaces of 24-well plates with an Elplasia round bottom plate-Ultra Low Attachment surface with a diameter of 500/700 μm (Corning, New York, USA) at a density of 35 × 10^4^ cells/well. The cells were suspended in EGM supplemented with 20% FBS at 37 °C and 5% CO_2_. After 2 days of aggregation, the cells were maintained under the same culture conditions for more than 1 month.

### Spheroid dissociation and flow cytometry analysis

For flow cytometry analysis, the dissociation of spheroids into single-cell suspensions was performed using an Embryoid Body Dissociation Kit (Miltenyi Biotec, Bologna, Italy) following the manufacturer’s instructions.

Monoclonal antibodies (mAbs) were used for flow cytometry (FACS) analysis. The antibodies used for the surface markers were as follows: CD133/2-PE human (1:100, REA820), CD24-APC (1:50, REA832), and anti-SSEA-4-FITC-human (1:50, REA101). The antibodies used for the detection of intracellular markers were as follows: anti-SOX2-FITC human and mouse (1:50, REA320); anti-OCT3/4-PE human and mouse (1:50, REA622); GATA 3, anti-human/mouse-APC (1:50, REA174); and anti-NANOG-APC human (1:50, REA314), REA Control Antibody, and human IgG1 (1:50, REA293). All mAbs were purchased from Miltenyi Biotec (Bologna, Italy). Intracellular staining was preceded by fixation and permeabilization with a Foxp3/Transcription Factor Staining Buffer Set (Thermo Fisher Scientific, Waltham, USA) before continuing with conjugated antibody staining. Adult resident stem/progenitor cells were incubated for 1 hour with cell membrane-specific antibodies in the dark at room temperature (RT). After surface staining, 100 µL of freshly prepared fixation/permeabilization working solution was added to each sample, and the mixture was pulse vortexed and incubated at RT for 40 minutes in the dark. The cells were washed with 1 mL of permeabilization buffer. Antibodies against intracellular markers were added to 100 µL of permeabilization buffer, incubated at RT for 1 hour in the dark, and then washed with 1 × PBS for acquisition. The area of positivity was determined by using an isotype-matched mAb, and in total, 10^4^ events were acquired for each sample. Five independent experiments were performed. The data were obtained by using an FC500 flow cytometer (Beckman Coulter, Brea, USA) and analyzed with Kaluza software (Beckman Coulter).

### Whole-mount and frozen section immunostaining

For whole-mount immunofluorescence experiments, spheroid/tube-like structures were fixed with 4% paraformaldehyde for 1 hour at RT and then fixed and permeabilized using blocking solution (3% BSA, 0.5% Triton X-100 in 1X PBS) for 2 hours at RT. The cells were incubated with primary antibodies in blocking buffer (1% BSA and 0.1% Triton X-100 in 1 × PBS) for 1 hour at RT. After the washes, the samples were incubated with secondary antibodies for 1 hour in blocking buffer solution (1% BSA and 0.1% Triton X-100 in 1X PBS). The following primary antibodies were used in this study: ZO-1 monoclonal antibody (R40.76-sc-33725: 1:200, rat monoclonal antibody, Santa Cruz Biotechnology, USA), CD13 monoclonal antibody (1C7D7: 1:200, Mouse/IgG1, Thermo Fisher Scientific, Waltham, USA), CD249 monoclonal antibody (OTI4G8:1:100, Mouse/IgG2b, Thermo Fisher Scientific, Waltham, USA), UROMODULIN polyclonal antibody (bs-2189R, 1:100, rabbit IgG, Bioss Antibodies, Woburn, USA), and LOTUS TETRAGONOLOBUS fluorescein (FITC, 10 μg/mL, Thermo Fisher Scientific, Waltham, USA). The secondary antibodies used were Alexa Fluor 488-conjugated goat anti-mouse IgG (1/200), Alexa Fluor 555-conjugated goat anti-mouse IgG (1/200), Alexa Fluor 488-conjugated goat anti-rabbit IgG (1/200), or Alexa Fluor 555-conjugated goat anti-rabbit IgG (1/200). All secondary antibodies were purchased from Thermo Fisher Scientific (Waltham, USA). Immunofluorescence staining of cryostat sections (4.5-µm thick) of spheroids and tubular-like structures was performed using an optimal shearing temperature (OCT) compound. Cryostat sections were washed with 1 × PBS and then labeled for 1 hour at RT. For the immunofluorescence staining of IgA1 deposits, a rabbit anti-human IgA (alpha chain) FITC-conjugated antibody (Diagnostic Biosystems, CA) was used on cryostat sections of ARPC-derived spheroids from IgAN patients and healthy subjects. Negative controls were prepared with irrelevant antibodies. Specimens were counterstained with 4′,6-diamidino-2-phenylindole (DAPI) (Sigma‒Aldrich, St. Louis, USA) and mounted in Fluoromount (Leica, Wetzlar, Germany). The stained cells were viewed under a Leica TCS SP5 confocal laser scanning microscope (Leica, Wetzlar, Germany) using ×20, ×40, and ×63 objective lenses.

### Labeling for in vitro and in vivo tracking

The cells were labeled with PKH-26 (Sigma-Aldrich, St. Louis, USA), an aliphatic red fluorescent chromophore. For labeling, ARPCs were dissociated by trypsin, centrifuged, washed, and incubated with PKH-26 according to the recommended protocol. The stained cells were viewed under a Leica TCS SP5 confocal laser scanning microscope (Leica, Wetzlar, Germany) using ×40 and ×63 objective lenses.

### Renin ELISA

Cell supernatants were collected on day 7 from both ARPCs in monolayers and from spheroids on day 10 after formation. The renin concentration was assessed using a Human Renin ELISA Kit (Thermo Fisher Scientific, Waltham, USA). Before performing the ELISAs, a total of 1 mL of cell supernatant from each condition was concentrated to 100 µL using Amicon Ultra 0.5 centrifugal filter devices (Merck Millipore, Darmstadt, Germany). In brief, the primary antibody was coated on the plate, and samples and standards were added to the wells for the reaction. Subsequently, a biotin-conjugated anti-human Renin antibody was added. After the reaction, streptavidin-HRP was added, and after washing away the unbound secondary antibody, tetramethylbenzidine was added to the wells, after which the color developed. The optical density was measured at 450 nm by a Thermo Scientific Multiskan FC Microplate Photometer. The obtained results were normalized both for the number of cells for each condition and for the quantity of the starting cellular supernatant.

### In vivo chorioallantoic membrane angiogenesis assay

Fertilized White Leghorn/Isa Brown chicken eggs obtained from a commercial supplier staged according to Hamburger and Hamilton (HH) were first placed in an incubator and kept under constant humidity at 37 °C (day (D) 0). At stage HH3 (D3), a square window was opened into the eggshell after the removal of 2-3 mL of albumen so that the developing chorioallantoic membrane (CAM) was detached from the shell itself and the underlying CAM vessels were observed. The window was sealed with a glass coverslip, and the eggs were returned to the incubator until the day of the experiment.

At D10 of CAM development, the top of the growing CAMs was visible, and the endothelium exhibited an intrinsically high mitotic rate. The coverslips were removed, and an 8 × 2 mm silicone ring was implanted near the vessel branch. Approximately 10 replicates of 1 × 10^6^ ARPC or RPTEC cells were obtained, and the ARPC-derived spheroids were resuspended in Matrigel and subsequently transplanted into the ring. The same volume of PBS was used as a negative control. The transplanted eggs were returned to the incubator for the next 4 days. At D14, the eggs were used for the experimental determinations.

Matrigel grafts with surrounding CAMs were fixed with 4% paraformaldehyde for 24 hours. CAMs were examined at D14 and photographed in vivo with a stereomicroscope equipped with a digital camera. The angiogenic response was evaluated by the IKOSA CAM Assay Application image analyzer system as the total length, area, and number of vessel branching points in the area surrounding the rings. Successively, the fixed CAMs were embedded in paraffin. Serial 4-μm sections were stained with hematoxylin and eosin. The slides were digitally scanned using an Aperio scan to evaluate the morphology of the vessels and the presence of transplanted cells.

### Statistical analyses

We analyzed data with statistical software GraphPad Prism (GraphPad, San Diego, CA, USA). All results are expressed as mean ± SEM. All values are expressed as the mean of data obtained from at least 3 independent experiments. Two-tailed Student’s *t*-test has been used to assess differences in biological features between 2 mean values.

## Results

### Human adult resident stem/progenitor cells generate spheroids and kidney tubular-like structures

**Figure 1.**
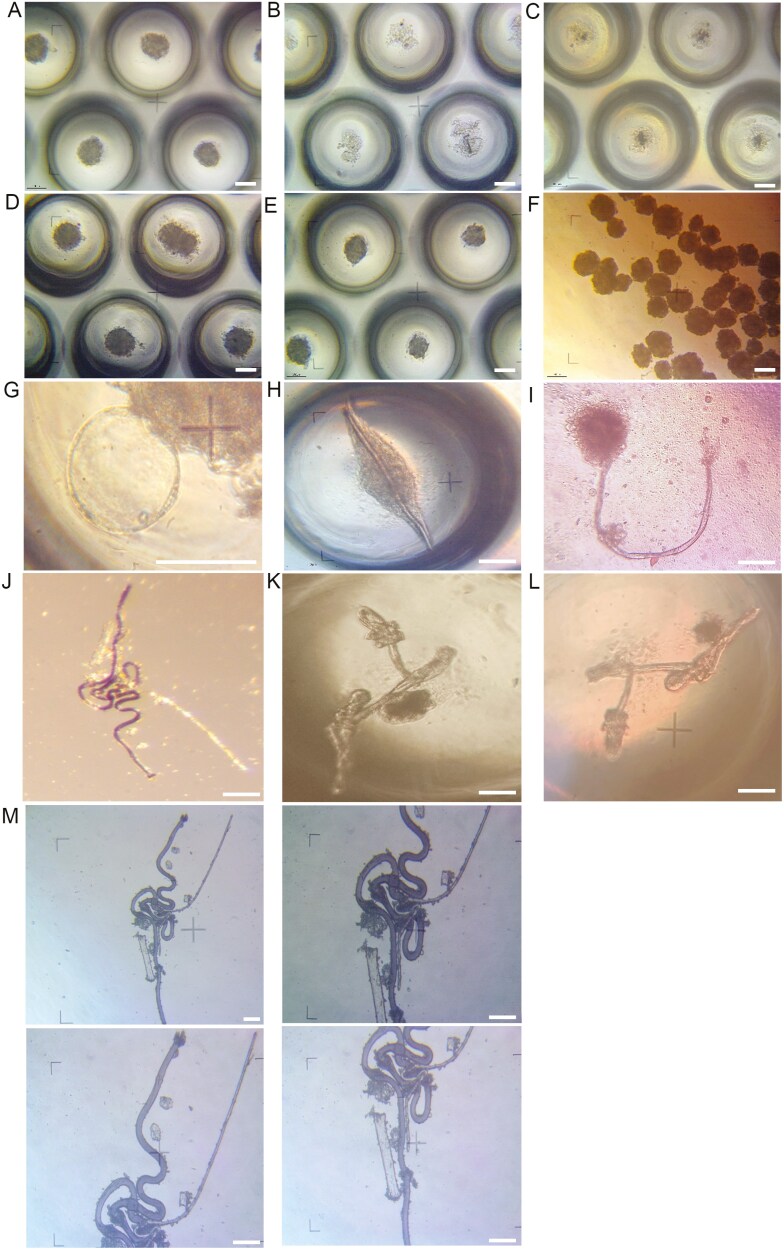
Generation of renal spheroids and tubule-like structures from ARPCs. (A) ARPCs formed spheroids after 2 days in 3D culture conditions. Each circle in the image represents the microcavity of the 3D plates containing a spheroid. (B) Primary and (C) immortalized RPTECs cannot form spheroids in 3D plates, and they appear to break up and disperse in the microcavities. Both tARPC (D) and gARPC (E) were able to form spheroids. (F) Spheroids derived from the mixed-cell population of uARPCs/CD133^-^ cells cultured in normal plates for 30-45 days without changing their morphology. (G) Spheroids from gARPCs formed circular protrusions similar to those of podocyte cell bodies connected by primary processes. (H-I) Spheroids derived from the mixed-cell population of uARPCs/CD133^-^ cells spontaneously generated tubular-like structures starting from one or both poles. (J-M) Tubule-like structures spontaneously generated by the uARPCs/CD133^-^ mixed cell population showing many similarities with the nephron unit of the kidney. (J) Image of a tubule-like structure acquired with a stereomicroscope. (M) Images acquired by a phase contrast microscope of the same tubule-like structure observed in (J) via a stereomicroscope. The scale bars represent 100 μm.

We isolated ARPCs, as previously described, from portions of the normal cortex of patients who underwent nephrectomy^13,14,16,46,47^ or from healthy patient urine (uARPCs).^44,45^ We confirmed that all ARPCs, including uARPCs, coexpressed CD133 and CD24 (Supplementary Figure S1). To investigate whether a limited microspace could promote cellular self-organization and regulatory mechanisms for the differentiation in typical organ structures, we plated 35 × 10^4^ cells in 3D plates under normal culture conditions. We observed that after only 2 days in 3D culture conditions, the ARPCs spontaneously aggregated and formed spheroids in the microcavities of the 3D plates (Figure 1A). The spheroids appeared compact and well aggregated, with a diameter of ~100 µm.

Spheroids were also generated from a mixed population of CD133^-^ renal cells and ARPCs, which accounted for 15%-50% of the total cells. To demonstrate that the stemness property of ARPCs is necessary for the formation of spheroidal aggregates, we seeded both primary and immortalized human RPTECs in 3D plates. Both were unable to aggregate and maintain a spheroidal conformation. In fact, after sowing they appeared to break up and disperse in the microcavities (Figure 1B-C). When we separately plated tubular ARPCs (tARPCs) and glomerular ARPCs (gARPCs), we found that both subpopulations were able to form spheroids (Figure 1D-E). Spheroids derived from uARPCs, tARPCs, gARPCs, or from the mixed cell population were stable, removed from 3D plates and cultured in normal plates for 30-45 days without changing their morphology (Figure 1F). The diameter of the spheroids ranged from 75 μm (5 days culture) to 160 μm (18-21 days culture). Some spheroids from gARPCs formed circular protrusions similar to those of podocyte cell bodies connected by primary processes (Figure 1G and Supplementary Figure S2A-C).

When spheroids were generated from a mixed cell population composed of 15%-50% of uARPCs or tARPCs and from CD133^-^ renal cells, the cells underwent morphological changes 6-12 days after formation, resulting in a widened, decreasing diameter extending along the axis. Without the addition of any external compounds, the spheroids were artificially modified in shape, and tubular-like structures were generated starting from one or both poles (Figure 1H-I and Supplementary Figure S2D-G). These structures had diameters ranging from 10 to 40 μm and could be very long, up to 700 μm. In some cases, the tubule-like structures could form structurally complex shapes that shared many similarities with the nephron unit of the kidney, such as the distal convoluted tubule, the loop of Henle, and the proximal convoluted tubule (Figure 1J-M). The tubule-like structures were formed by about 35% of spheroids, whereas the circular protrusions were formed by about 10% of spheroids. The nephron-like structures shown in Figure 1J-M formed more rarely. The spheroids could be successfully expanded in low-attachment cell culture plates, at least for 4 passages.

To determine the origin of the tubular-like structures observed via optical microscopy, we labeled ARPCs and RPTECs with the PKH26 vital tracker, a lipophilic fluorescent probe useful for in vitro and in vivo cell tracking applications, before seeding them in 3D plates. We observed the structures via fluorescence microscopy after 4 and 10 days of 3D culture. The spheroids generated from ARPCs were dense with a thick edge (Supplementary Figure S3A), whereas the spheroids from RPTECs appeared disaggregated without a boundary defined by PKH26 (Supplementary Figure S3B). The tracker allowed us to demonstrate the origin of long tubule-like structures from spheroids (Supplementary Figure S3C-E). Any tubular structures were not generated by the RPTECs. We did not observe differences in tubular-like structures generated from uARPCs or tARPCs.

### ARPC spheroids express typical markers of pluripotent cells

**Figure 2.**
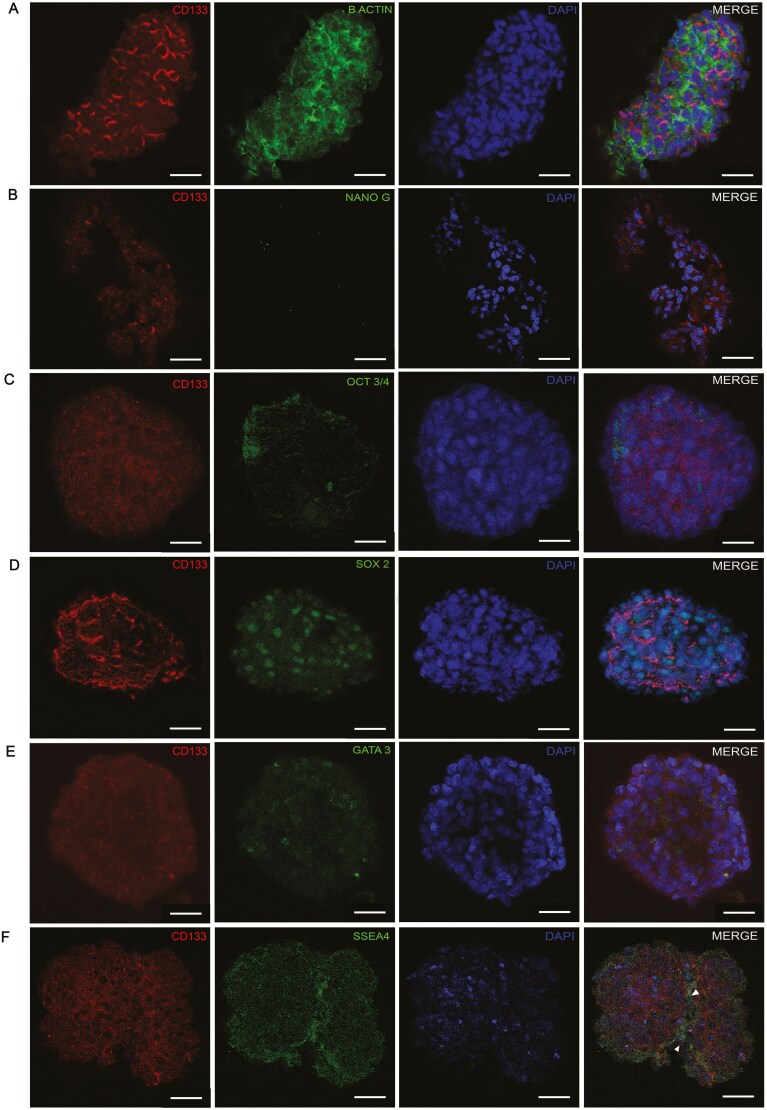
Expression of stem cell markers in ARPC spheroids. (A-C) Whole-mount double-label immunofluorescence shows that uARPC spheroids expressed high levels of the functional and constitutional marker ARPC CD133. The spheroids were also stained with beta-actin to determine their structure and integrity. Spheroids expressed low levels of the transcription factors NanoG (B) and Oct3/4 (C) and elevated levels of the SOX2 (D), GATA-3 transcription factors (E), and of the embryonic stem cell marker SSEA4 (F). Spheroids coexpressing CD133/SSEA4 can spontaneously form the typical groove of organoids (F, arrows). The scale bars represent 25 μm.

The 3D structures of the uARPC spheroids and tARPCs spheroids were characterized via whole-mount immunofluorescence staining, which was used to detect the expression of stem cell markers 5 days after formation. Specifically, spheroids expressed high levels of CD133, the functional and constitutional marker of ARPCs. The spheroids were also stained with beta-actin to determine their structure and integrity (Figure 2A). Furthermore, we showed that the spheroids expressed very low levels of transcription factors typical of embryonic stem cells, such as NanoG and Oct3/4 (Figure 2B and C). However, ARPC-derived spheroids expressed SOX2 (Figure 2D) and GATA-3 (Figure 2E), which are typical stem cell transcription factors, and stage-specific embryonic antigen 4 (SSEA4; Figure 2F), a glycoprotein expressed during early development and a marker of human embryonic stem cells.^49^ In some cases, spheroids coexpressing CD133/SSEA4 spontaneously formed the typical groove of organoids (Figure 2F, arrows). In addition, we found that spheroids were also positive for PAX2, another important stemness marker of ARPCs (Supplementary Figure S4).

### Cytofluorimetric analysis showing stem cell marker expression

**Figure 3.**
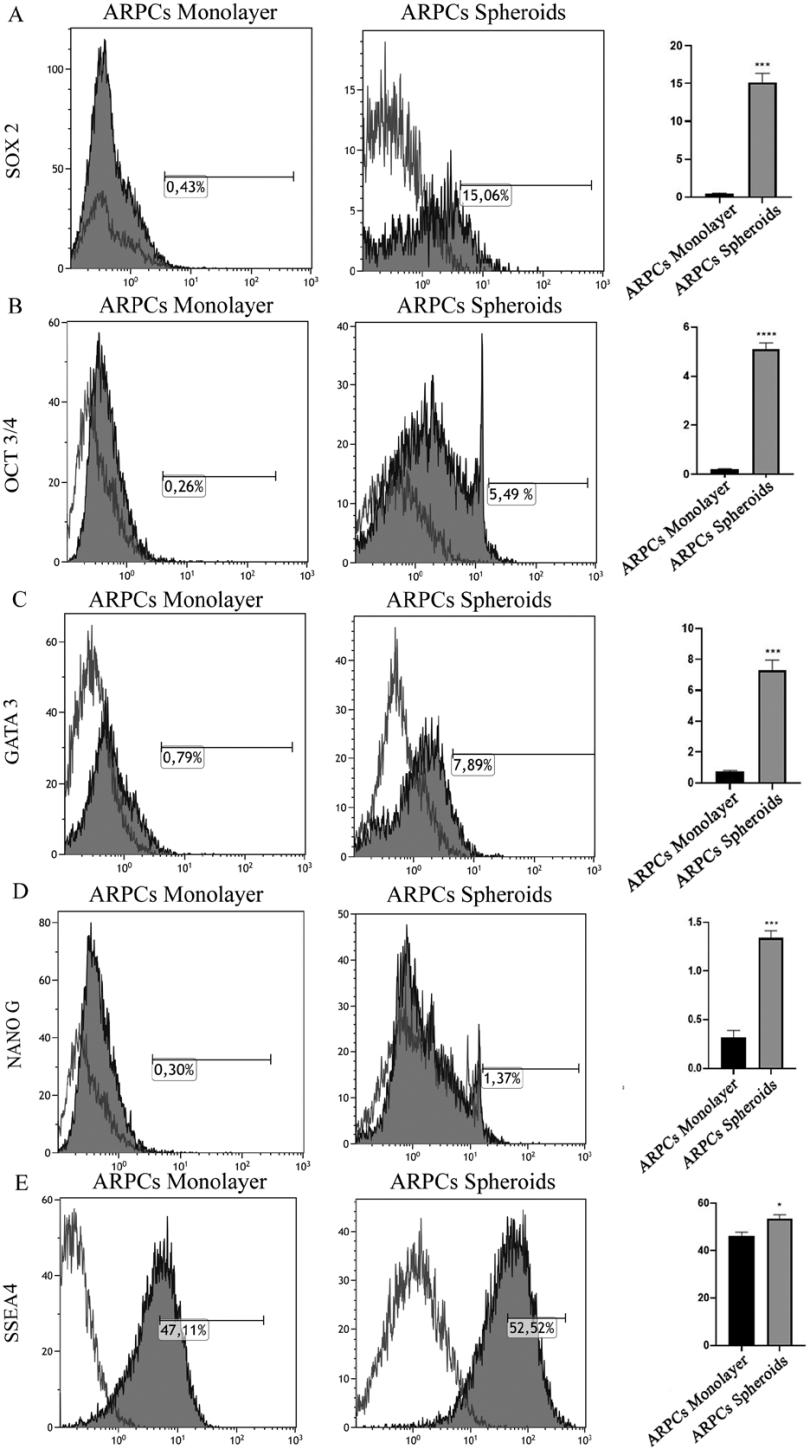
Cytofluorometric analysis showing the expression of stem cell markers in the uARPCs/CD133^−^ mixed cell population and in cells derived from spheroids. (A-C) Compared with monolayer ARPCs, spheroids expressed higher levels of SOX2, OCT3/4, and GATA3. (D) NanoG was not expressed or was expressed at low levels in the uARPCs/CD133^-^ mixed cell population or in spheroids. (E) Both the uARPC/CD133^-^ mixed cell population in the monolayer and cells from spheroids expressed high levels of the SSEA-4 marker. For each analyzed marker, the percentage data from triplicate samples are shown in histograms as mean ± SEM. *P <.05; ***P <.0005, ****P <.0001.

To investigate whether stem cell marker expression differences were present in spheroids generated from the uARPCs/CD133^-^ mixed cell population or from the same cell population in monolayer culture, we analyzed by FACS cells disaggregated from spheroids formed after 5 days and analyzed the ARPC counterparts in monolayers. We found that, in spheroids, there was a greater expression of SOX2, OCT3/4, and GATA3 than in monolayer ARPCs (Figure 3A-C). However, NanoG resulted in no or low expression (Figure 3D). Interestingly, both ARPC in monolayer and cells from spheroids exhibited high levels of the SSEA-4 marker, which, to date, was not known to be expressed in ARPCs (Figure 3E).

### Tubular-like structures expressed markers typical of renal tubules

**Figure 4.**
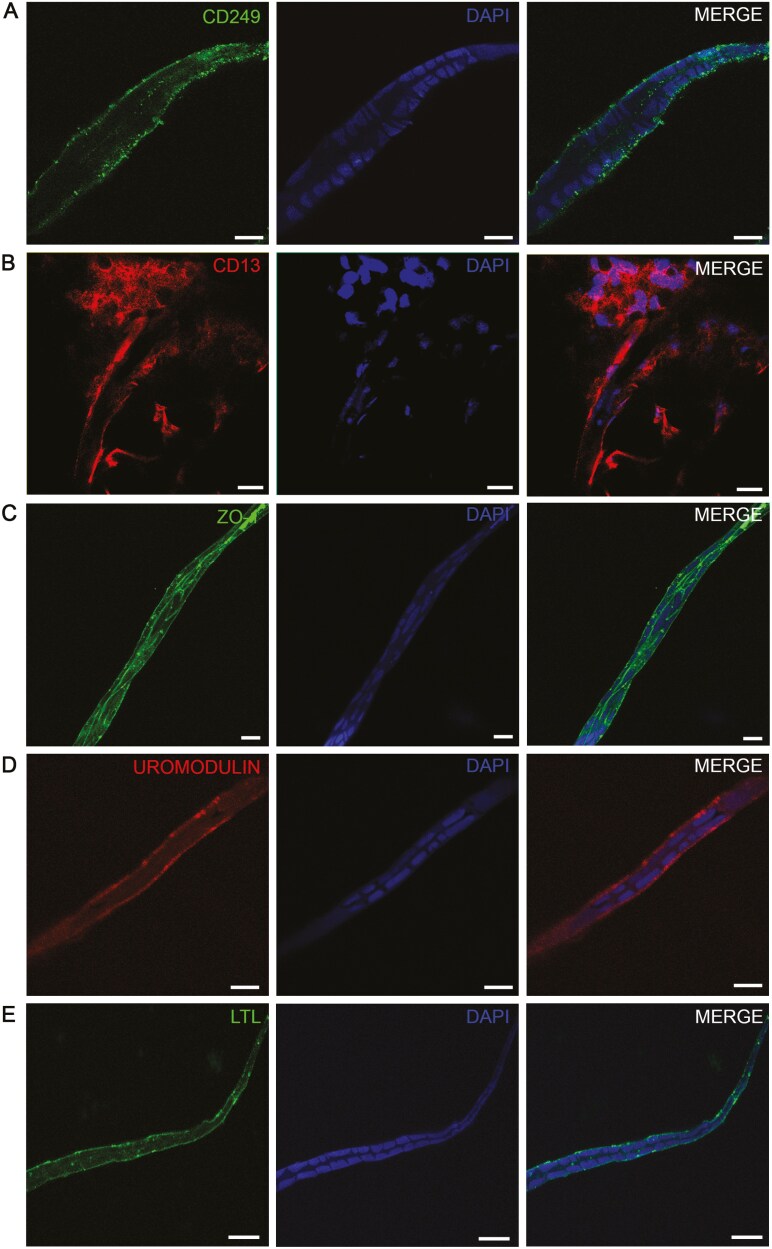
Expression of renal tubular markers in tubular-like structures. (A) Whole-mount immunofluorescence showing the expression of CD249 in tubular-like structures. (B) Whole-mount immunofluorescence showing that the tubule-like structures generated from spheroids were positive for CD13 (aminopontinidase N). (C) Whole-mount immunofluorescence showing the expression of ZO-1 in tubular-like structures. (D) Whole-mount immunofluorescence showing the expression of uromodulin in tubule-like structures generated from spheroids. (E) Whole-mount immunofluorescence showing the expression of lotus tetragonolobus lectin in some tubular-like segments. The scale bars represent 15 μm in A, and 25 μm in B-E. Cell nuclei were visualized using DAPI.

We performed whole-mount immunofluorescence staining of tubular-like structures originating from uARPCs and tARPCs spheroids after 15 days. Typical renal tubular markers, such as CD249 (glutamyl aminopeptidase or aminopeptidase A), CD13 (aminopeptidase N), ZO-1, Uromodulin, and LTL, were used. CD249 is an aminopeptidase A enzyme involved in the renin-angiotensin system, and its expression was rather uniform at the edges of tubular-like structures (Figure 4A).

We also characterized tubules for CD13, which is usually concentrated in the membrane of renal PT cells. The tubule-like structures generated from spheroids were positive for this marker (Figure 4B). It was expressed in long portions of tubular structures (Supplementary Figure S5A), even if it was not uniform (Supplementary Figure S5B). To characterize the apical intercellular junctional complex in tubular-like structures, we performed immunofluorescent experiments with the epithelial marker ZO-1. It was expressed uniformly throughout the lumen and at the outer borders of all long tubule-like segments (Figure 4C and Supplementary Figure S5C). Importantly, after tubular-like differentiation, we also observed strong positive staining for Uromodulin, a marker usually localized in the epithelial cells of the ascending limb of the loop of Henle (Figure 4D). In Supplementary Figure S5D, it is possible to observe a tubule that comes out of the spheroid.

The identity of the tubular-like structures was also probed using LTL, which binds to glycoproteins present on the surface of proximal tubules (Figure 4E). Some tubular segments elongated from uARPCs and tARPCs spheroids formed loop structures (Supplementary Figure S5E).

**Figure 5.**
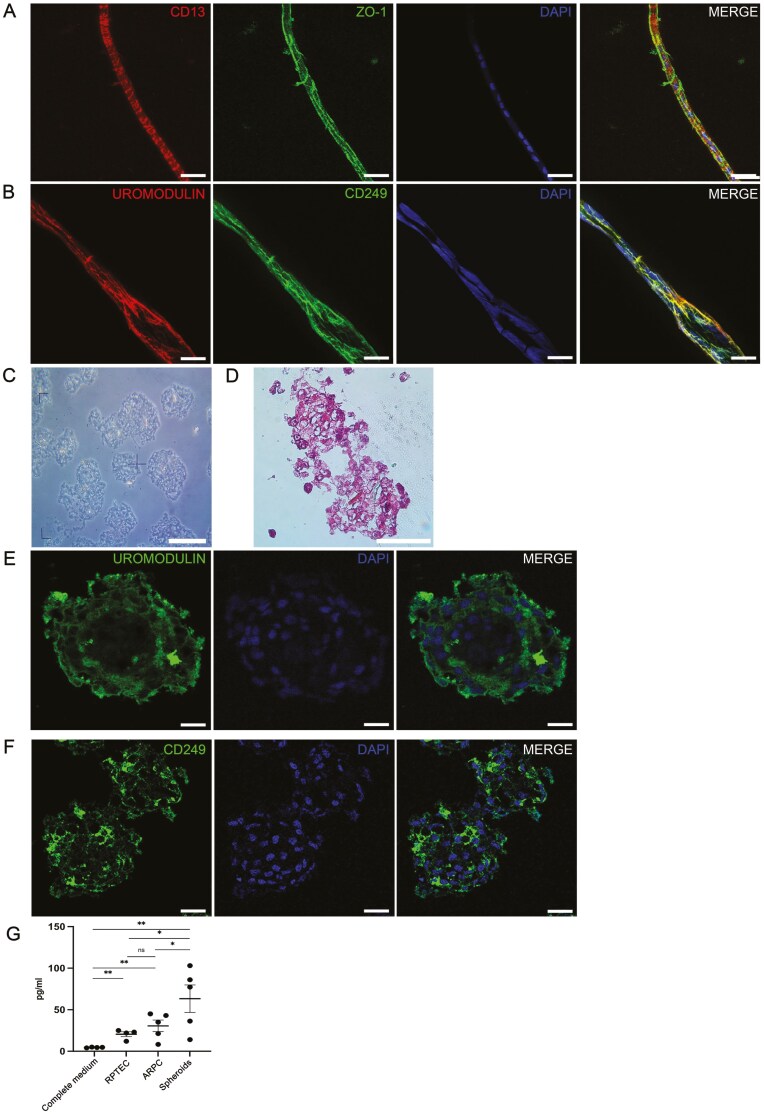
Expression of renal tubule markers in tubular-like structures generated by ARPC spheroids. (A) Whole-mount double-label immunofluorescence showing the coexpression of CD13 with ZO-1. (B) Whole-mount double-label immunofluorescence showing the coexpression of uromodulin with lotus tetragonolobus lectin. (C) Optical microscope image of spheroid sections. (D) Hematoxylin–eosin staining of spheroid sections. (E) Immunofluorescence image showing uromodulin in spheroids and sections with a tubule-like structure. (F) Immunofluorescence showing the expression of CD249 in spheroids and tubule-like structure sections. Nuclei were counterstained with DAPI. The scale bars represent 30 μm in A, 20 μm in B and E-F, and 100 μm in C and D. (G) ELISA showing that after 10 days of culture, ARPC-derived spheroids had elevated levels of renin compared with those in RPTECs and compared with those in ARPC monolayers. *P <.05; **P <.005.

Some of the tubule-like segments also coexpressed ZO-1 and CD13 (Figure 5A) or expressed uromodulin and CD249 (Figure 5B).

We also performed immunofluorescence staining of uromodulin on OCT-embedded samples of ARPC spheroids and tubule-like structures (Figure 5C-D). We confirmed that they were positive for uromodulin (Figure5E) and CD249 (Figure5F).

### ARPC-derived spheroids secreted elevated levels of renin

We then investigated whether spheroids and tubular-like structures also differentiated functionally in a more complex mature renal system. We measured the levels of renin, the angiotensinogenase enzyme that participates in the body’s renin–angiotensin–aldosterone system, in RPTECs, ARPCs, and spheroids after 10 days of culture. We found that both RPTECs and ARPCs in monolayer culture secreted renin and that the amount of renin produced by ARPCs was slightly greater than that produced by RPTECs (30 and 23 pg/mL, respectively; Figure 5G). However, compared with both ARPC and RPTEC monolayers, renal spheroids produced and secreted significantly more renin (63 pg/mL, *P* <.05; Figure 5G).

### ARPCs and ARPC-derived spheroids showed angiogenic properties

**Figure 6.**
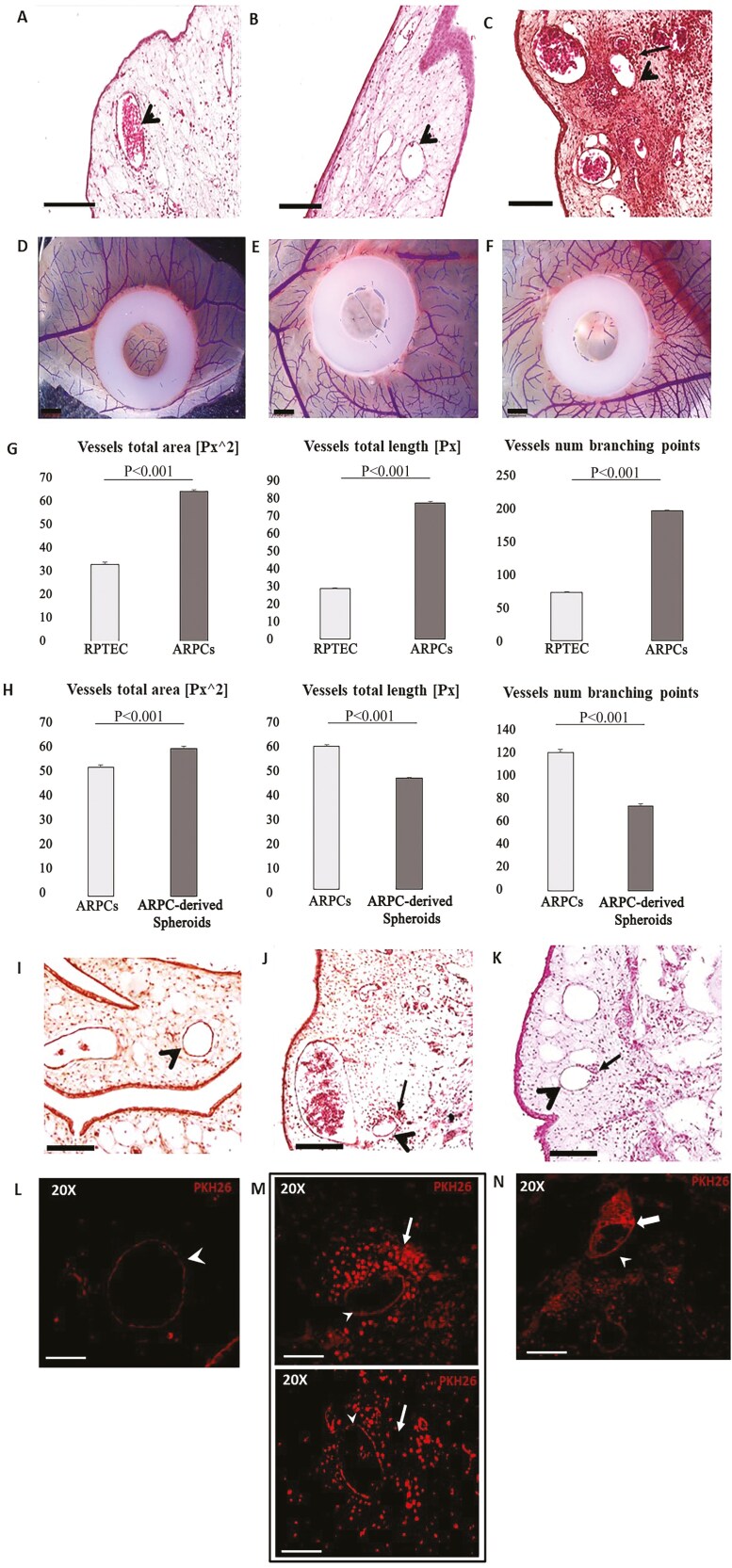
Angiogenic properties of ARPC and ARPC-derived spheroids. Microscopic (A-C) and macroscopic (D-F) images of CAMs transplanted with a silicon ring alone (A, D) or with a silicon ring containing inside RPTECs (B, E) and ARPCs (C, F). The A-C sections are H&E stained; the arrowheads indicate some vessels and the arrow in C a group of ARPCs cells near the vessel. In G, histograms showing the postimage analysis of vessel total area, vessel total length, and number of vessel branching points, expressed as percent variation in RPTECs and ARPCs compared with the control (silicon ring alone). (H) Histograms showing the postimage analysis of vessel total area, vessel total length, and number of vessel branching points, expressed as percent variation in ARPCs and ARPC-derived spheroids compared with controls. (I-K) Microscopy images of CAMs transplanted with a silicon ring alone (control, I) or a silicon ring containing inside ARPCs (J) or ARPC-derived spheroids (K). The arrowheads indicate some CAM vessels and the arrows in J and K indicate a group of ARPCs and ARPC-derived spheroids, respectively, near the vessels. (L-N) Fluorescence microscopy images of a CAM vessel (arrowhead) in a control (L), in PKH26-labeled ARPC-transplanted CAM vessels (arrowhead, M) and in CAM vessels (arrowhead) transplanted with spheroids generated from PKH26-labeled ARPCs. The arrows in (M) indicate the ARPCs around the vessels. The arrows in (N) indicate the spheroids near the vessel. Scale bar: A-K 100 μm; L-N 25 μm.

To investigate whether spheroids had angiogenic properties, we performed chick CAM experiments to compare angiogenesis induced by spheroids, ARPCs and RPTECs via in vivo assays (Figure 6A-E). We found that, with respect to conditions without transplanted cells (Figure 6A, D), ARPC- and spheroid-transplanted CAMs (Figure 6C, F) induced a stronger angiogenic response than RPTEC-transplanted CAMs (Figure 6B, E). Postimaging analysis of the percent variation in vessel total area (VA), vessel total length (VL), and number of vessel branching points (BP) revealed significant differences in CAMs transplanted with ARPCs (VA: 64 ± 0.7; VL: 78 ± 0.7; BP: 199 ± 1.33) compared with those transplanted with RPTEC (VA: 33 ± 0.9; VL: 30 ± 0.6; BP: 74 ± 1) (Figure 6G). When we compared CAMs transplanted with ARPC-derived spheroids and those transplanted with ARPCs, we found greater increases in the angiogenic VL and BP parameters in CAMs transplanted with ARPCs (VL: 60 ± 0.8; BP:120 ± 2.3) than in those transplanted with spheroids (VL: 47 ± 0.5; BP:74 ± 2.3). Conversely, compared with those in ARPC transplants, VA in spheroids was greater (VA: 60 ± 0.2 vs 53 ± 0.2, respectively) (Figure 6H). Hematoxylin–eosin sections from CAMs transplanted with a silicon ring alone (control; Figure 6I), a silicon ring containing inside ARPCs (Figure 6J) or containing ARPC-derived spheroids (Figure 6K) showed more cells near the vessels in the ARPCs than in the control. However, few spheroid-derived cells were observed near the vessels (Figure 6K). To confirm these observations, we performed new CAM experiments using cells stained with the cell tracker PKH26 (Figure 6L-N). We confirmed the presence of a high concentration of fluorescent ARPCs near the CAM vessels (Figure 6M). CAMs transplanted with ARPC-derived spheroids showed the presence of some spheroids near the vessels (Figure 6N). Few labeled spheroid-derived cells were observed (Figure 6N).

### ARPCs isolated from the urine of IgA nephropathy patients formed renal spheroids capable of recapitulating the characteristic IgA1 deposition observed in this disease

**Figure 7.**
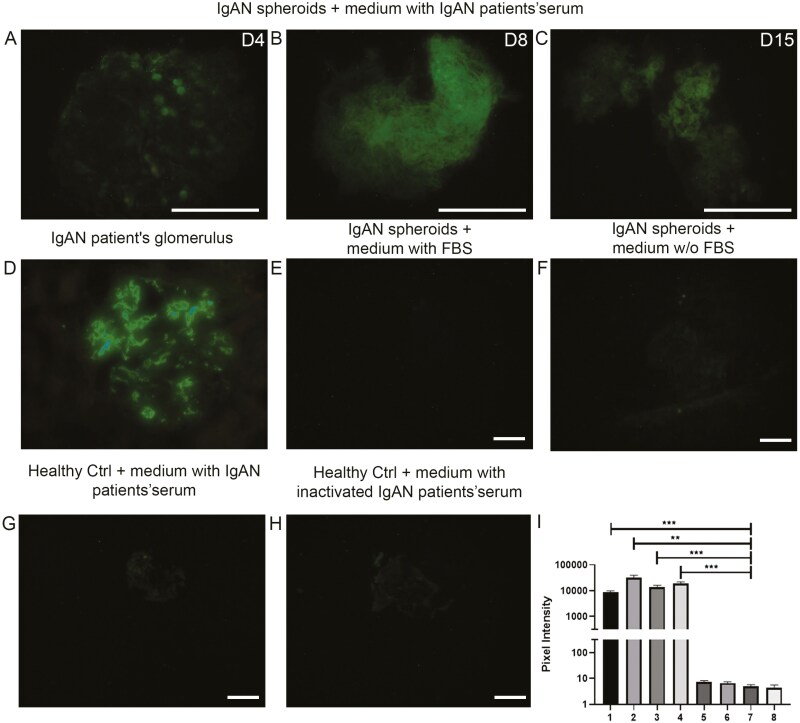
Immunofluorescence staining of IgA1 deposits in ARPC-derived spheroids from IgAN patients and healthy subjects. ARPC-derived IgAN spheroids exhibited IgA1 deposits 4 days (A), 8 days (B), and 15 days (C) after the addition of IgAN patient serum. (D) IgA1 deposition in the glomerulus of a patient diagnosed with IgAN. (E) IgA1 immunostaining of IgAN spheroids cultured with FBS for 15 days. (F) IgA1 immunostaining of IgAN spheroids cultured without FBS for 15 days. (G) IgA1 immunostaining of ARPC-derived spheroids from healthy controls cultured with IgAN patient serum for 15 days. (H) IgA1 immunostaining of ARPC-derived spheroids from healthy controls cultured with inactivated IgAN patient serum for 15 days. (I) Fluorescence levels of IgA1 deposits in IgAN spheroids cultured with IgAN patient serum for 4 days (1), 8 days (2), or 15 days (3); in IgAN patient glomeruli (4); in IgAN spheroids cultured with FBS (5) or without FBS (6); in healthy subjects with inactivated IgAN patient serum (7); or in those cultured with IgAN patient serum (8). The scale bars represent 50 μm in A-C and 25 μm in E-H. Results are representative of 3 independent experiments (3 different IgAN patients). The immunofluorescent quantification from triplicate samples is shown in the histogram as mean ± SEM. **P <.01; ***P <.001.

To study the proof of concept that ARPC-derived spheroids can be used to establish in vitro models recapitulating renal diseases, we isolated ARPCs from the urine of IgA nephropathy (IgAN) patients and generated patient-specific renal spheroids using the ARPC mixed cell population method. We checked whether the spheroids expressed glomerular markers such as nephrin and CD2AP and we found positivity after 7 days of culture (Supplementary Figure S6). Then, we performed culture experiments with IgAN patient serum. After a 3-day incubation period, the medium containing FBS was removed, except for the medium from the control group. Subsequently, the spheroids were cultured for 15 days in the presence of active or inactivated IgAN patient serum. The same experiments were performed using samples from healthy controls. Then, we checked for the presence of IgA1 deposits on spheroids with the same antibody utilized for the clinical diagnosis of IgAN by utilizing immunofluorescence staining of biopsy renal tissue. We found that the ARPC-derived spheroids of IgAN patients already showed IgA1 deposits after 4 days of culture in patient serum (Figure 7A) and exhibited a different pattern after 8 days (Figure 7B) and 15 days (Figure 7C). The IgA1 deposition resembled that observed in the glomeruli of patients with IgAN (Figure 7D). In contrast, no positive signal was detected in either IgAN spheroids cultured in FBS (Figure 7E) or in the control without serum (Figure 7F). Additionally, spheroids derived from the urine of healthy controls cultured in IgAN serum (Figure 7G) or inactivated IgAN serum (Figure 7H) did not show IgA1 deposits. The fluorescence intensity of IgA1 was comparable between deposits in spheroids and in IgAN patient glomeruli (Figure 7I).

## Discussion

The results of this study demonstrated the potential of human adult renal stem/progenitor cells (ARPCs) to form spheroids and artlessly generate tubular-like structures in vitro. These structures exhibited characteristics of renal tubules and expressed markers associated with renal progenitor cells and pluripotent cells. Notably, the generation of these tubular-like structures was driven solely by the ARPCs themselves, without the need for external chemokines or growth factors. This unique characteristic of these cells is novel and contrasts with the current methods of generating renal organoids and tubuloids, which rely on very complex differentiation protocols and the external administration of growth factors.

The formation of spheroids by ARPCs suggested inherent self-organization and regulatory mechanisms, facilitating their differentiation into typical organ structures. This capacity of ARPCs to aggregate and spontaneously differentiate into tubule-like structures underscores their unique regenerative potential for applications in medicine.

Our study revealed that spheroids generated from a cell population comprising ARPCs and CD133^-^ cells cultured together for 5-10 days could give rise to elongated tubule-like structures resembling nephrons in certain instances. In contrast, RPTECs did not exhibit spheroid formation, indicating the indispensable role of the stemness of ARPCs in this process.

Moreover, we demonstrated the feasibility of generating spheroids and tubular structures directly from renal progenitors isolated from the urine of both healthy subjects and glomerulonephritis patients. This breakthrough opens promising avenues for further research in renal medicine.

The tubular-like structures generated from ARPC-derived spheroids exhibited characteristics of renal tubules, such as the expression of the renal tubule markers CD249, CD13, ZO-1, Uromodulin, and LTL. These markers are typically associated with different segments of renal tubules, indicating that the tubular-like structures generated from ARPCs have the potential to mimic the structure and function of various regions of the nephron. CD249 (glutamyl aminopeptidase or aminopeptidase A) is an enzyme that can regulate blood pressure by cleaving the N-terminal aspartate from angiotensin II, degrading vasoconstricting angiotensin II into angiotensin III. It is expressed by the epithelial cells of proximal tubules and the glomerulus of the nephron.^50^ CD13, also known as aminopeptidase N, is a membrane-bound protein that catalyzes the formation of the natriuretic hexapeptide angiotensin IV from angiotensin III. In the kidney, it is primarily present in the brush border membrane of proximal tubule cells, where it reduces basolateral Na^+^-K^+^-ATPase activity.^51,52^ The tight junction protein ZO-1 is expressed at cell boundaries independently of the identity of the tubule^53^ and can regulate proximal tubular cell differentiation.^54,55^ Some tubule-like structures were also positive for uromodulin or Tamm–Horsfall protein.

This protein is produced by the thick ascending limb of the loop of Henle of the mammalian kidney and represents the most abundant protein in normal human urine. The ectodomain of its glycosyl phosphatidylinosital-anchored counterpart, which is located on the luminal cell surface of the loop of Henle, is cleaved by proteases, leading to its excretion in urine. It is the matrix of urinary casts that is derived from the secretion of renal tubular cells.^56,57^ Some portions of the tubule-like structures were also positively stained with LTL, which binds selectively to renal proximal tubules.^58^

The expression of pluripotent cell markers such as CD133, PAX2, NanoG, Oct3/4, SOX2, and GATA-3 in ARPC-derived spheroids suggested that these structures may possess pluripotent properties. This is further supported by the high expression of stage-specific embryonic antigen 4 (SSEA4), a marker of human embryonic stem cells and of embryonic early development,^49,59^ which was, for the first time, observed in ARPCs or renal spheroids. The coexpression of CD133 and SSEA4 in some spheroids may indicate the presence of progenitor cells capable of differentiation and development into organoids.

Furthermore, cytofluorimetric analysis revealed greater expression of the stem cell markers SOX2, OCT3/4, and GATA3 in spheroids than in ARPCs in monolayer culture. These findings suggested that the 3D culture environment promotes the maintenance of stem cell properties in ARPCs. We showed that, compared with RPTECs and ARPCs, ARPC-derived spheroids cultured for 10 days also secreted high amounts of renin. Salt reabsorption, blood pressure, and volume homeostasis are regulated by this enzyme, which is generated by the adult kidney. Renin-producing cells, which are derived from a similar stromal progenitor population, can be found in the undifferentiated metanephric mesenchyme during kidney development and are widely distributed.^60^ These data are in agreement with previous studies showing that tubular cells can also produce renin^61-63^ and demonstrating that renin can be a hallmark of the functional maturity of renal organoids.^64^

In addition, we demonstrated the angiogenic properties of ARPCs and ARPC-derived spheroids through the use of CAM assays. Compared with those of the control group, the transplantation of ARPCs and spheroids onto the CAM resulted in an increased vessel area, a longer total length, and a greater number of branching points. These findings suggest that ARPCs and their derived spheroids can stimulate angiogenesis, which could be beneficial for vascular regeneration in renal tissue.

Our study also showed that ARPCs isolated from patients with IgA nephropathy formed spheroids that resembled the IgA1 deposition characteristic of the disease. Indeed, IgA1 deposits formed in uARPC spheroids isolated from IgAN patients following their culture with patient serum. These spheroids expressed also 2 typical markers of podocytes, nephrin, a component of the glomerular slit diaphragm, and CD2AP which is expressed with nephrin in developing podocytes and is found widely in the mature kidney as an adapter molecule that can bind to the cytoplasmic domain of nephrin. This highlights the potential of ARPC-based models for studying and understanding disease mechanisms, as well as developing personalized therapies for patients with specific renal disorders. Even if in this study we analyzed the principal pluripotent and tubular markers, a more comprehensive study of remaining pluripotent and nephron markers, including glomerular markers, in spheroids and tubule-like structures is needed.

Overall, this study provides insights into the regenerative capabilities of ARPCs and their potential for generating renal spheroids/organoids and spontaneous tubule-like structures in vitro. The ability of ARPCs to form spheroids and differentiate into tubular-like structures without the need for external chemokines or growth factors is a significant advancement in the field, especially considering that these cells can be isolated from the urine of patients. ARPC-derived spheroids also can be created easily and can expand in vitro naturally, allowing the possibility of creating spheroid biobanks for personalized medicine.^33,65^ These findings open up new opportunities for studying kidney development and disease mechanisms and developing regenerative therapies for renal disorders. However, further research is needed to fully characterize the functional properties of ARPC-derived structures and their potential applications in regenerative medicine.

## Supplementary Material

szaf002_suppl_Supplementary_Figures_1-6

## Data Availability

The data underlying this article are available in the article and in its online supplementary material.
