# Supplementary material for: Unveiling spontaneous renal tubule-like structures from human adult renal progenitor cell spheroids derived from urine
Source: Stem Cells Transl Med. 2025 Mar 29;14(4):szaf002. doi: 10.1093/stcltm/szaf002 (PMC11954590; doi:10.1093/stcltm/szaf002)
Supplement: szaf002_suppl_Supplementary_Figures_1-6 [file szaf002_suppl_supplementary_figures_1-6.pdf]

Unveiling Spontaneous Renal Tubule-Like Structures from Human Adult Renal Progenitor Cell Spheroids Derived from Urine

Francesca Giannuzzi, Angela Picerno, Silvia Maiullari, Francesca Montenegro, Antonella Cicirelli, Alessandra Stasi, Giuseppe De Palma, Vito Francesco Di Lorenzo, Giovanni Battista Pertosa, Paola Pontrelli, Michele Rossini, Nunzia Gallo, Luca Salvatore, Vincenzo Di Leo, Mariella Errede, Roberto Tamma, Domenico Ribatti, Loreto Gesualdo, Fabio Sallustio.

Supplementary Material

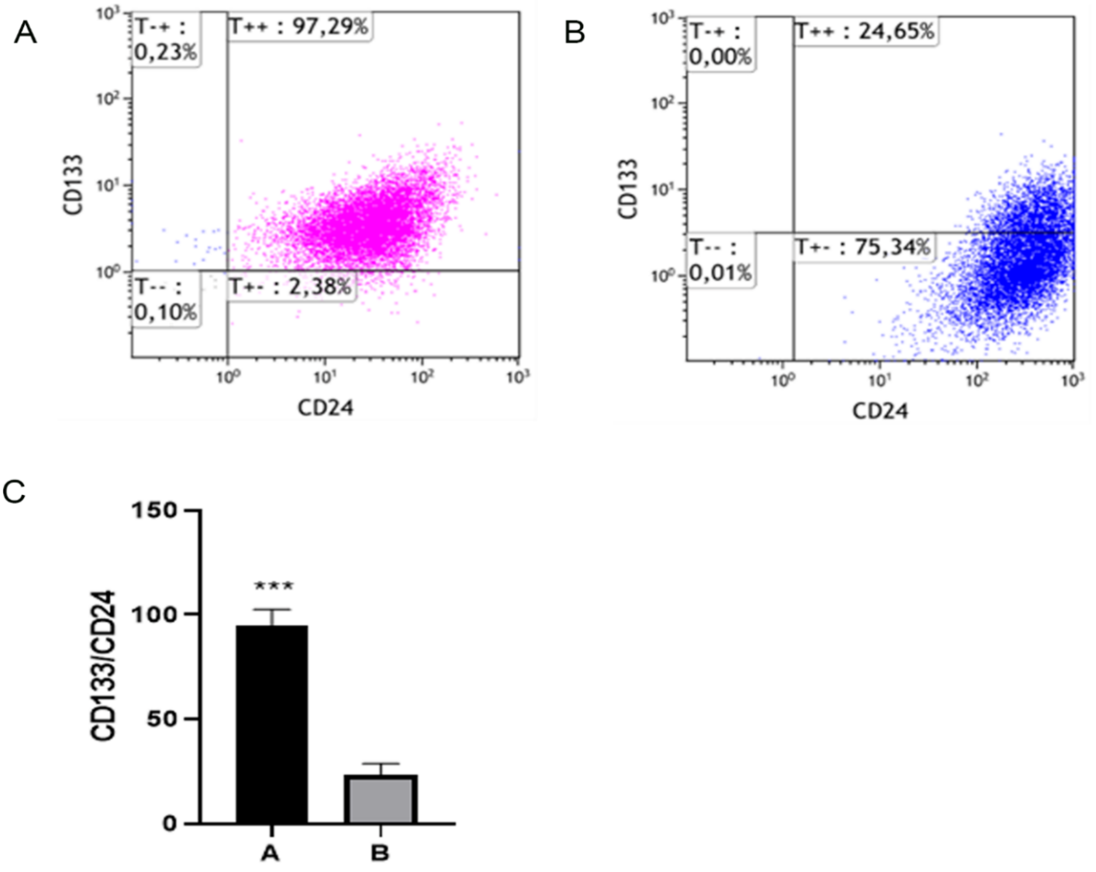

Supplemental Figure 1. Cytofluorimetric analysis showing the expression of CD133 and CD24.

(A) ARPCs isolated from urine of a healthy subject co-expressing high levels of CD133 and CD24 markers**.** **(B)** uARPCs/CD133$^-$ mixed cell population used to generate renal spheroids. **(C)** Histogram showing the percentage data from triplicate samples as mean±SEM of CD133$^+$/CD24$^+$ cells in cell populations isolated from urine of healthy subjects (A) and in mixed cell populations used to generate renal spheroids *p<0.05; *** p<0.0005.

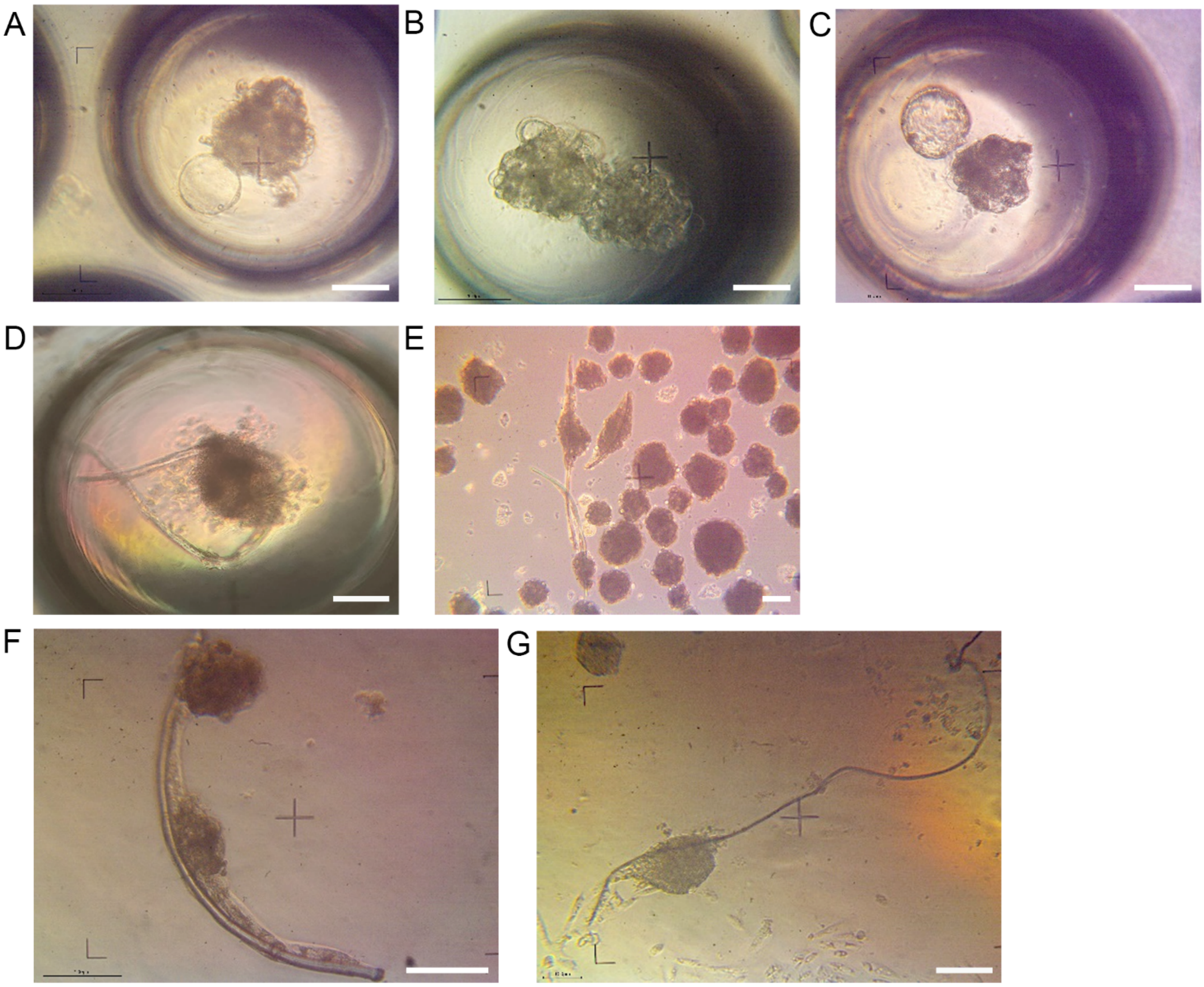

Supplemental Figure 2. Generation of renal spheroids and tubule-like structures from ARPCs. (A-C) Spheroids from gARPCs formed circular protrusions similar to those of podocyte cell bodies connected by primary processes. **(D-G)** Spheroids derived from the mixed-cell population of uARPCs/CD133- cells spontaneously generated tubular-like structures starting from one or both poles. The scale bars represent 100 μm.

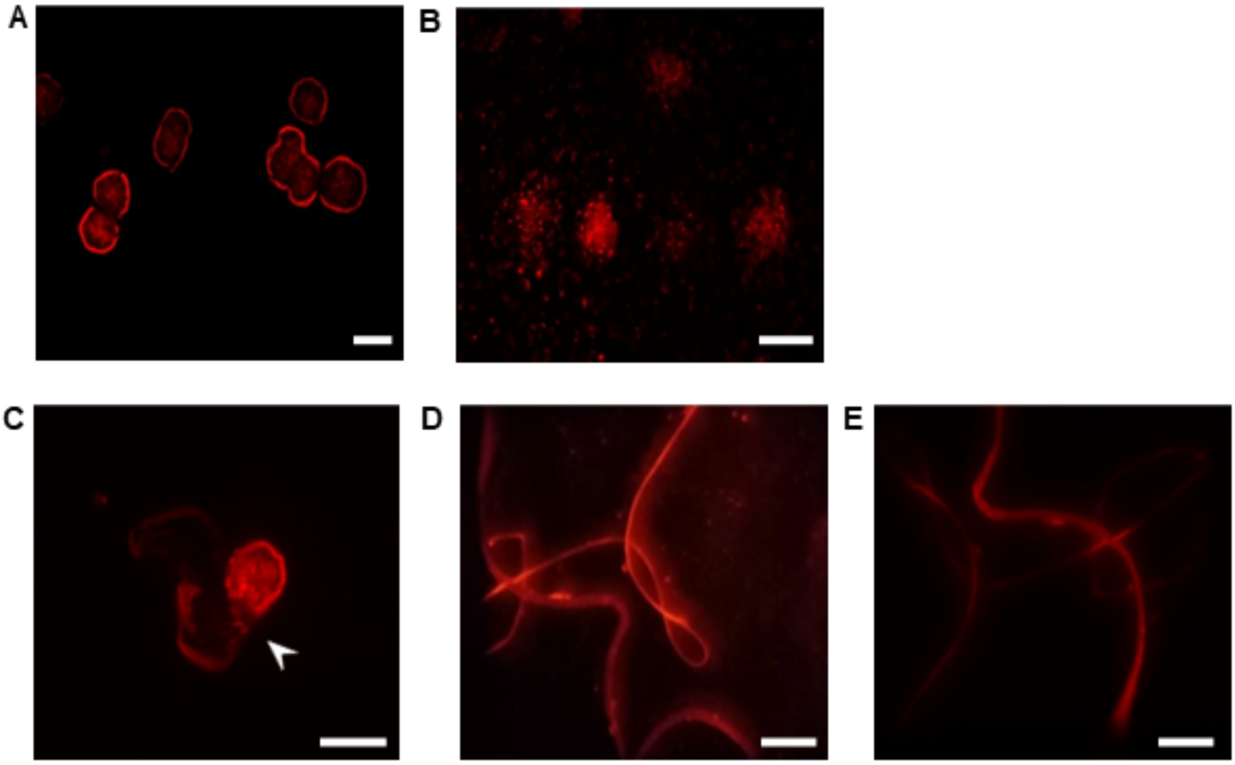

Supplemental Figure 3. Tracking of sprouting tubule-like structures from spheroids. ARPCs were labeled with the PKH26 fluorescent cell tracking marker and induced to form spheroids in 3D plates. The spheroids generated from the mixed uARPCs/CD133$^-$cell population were dense with clear and defined thick edges **(A),** whereas the spheroids generated from the RPTECs appeared to be disaggregated **(B). (C-E)** The fluorescent tracker demonstrated the origin of long tubule-like structures from ARPCs generating spheroids. The arrow indicates a tubule sprouting from a spheroid. The scale bars represent 100 µm.

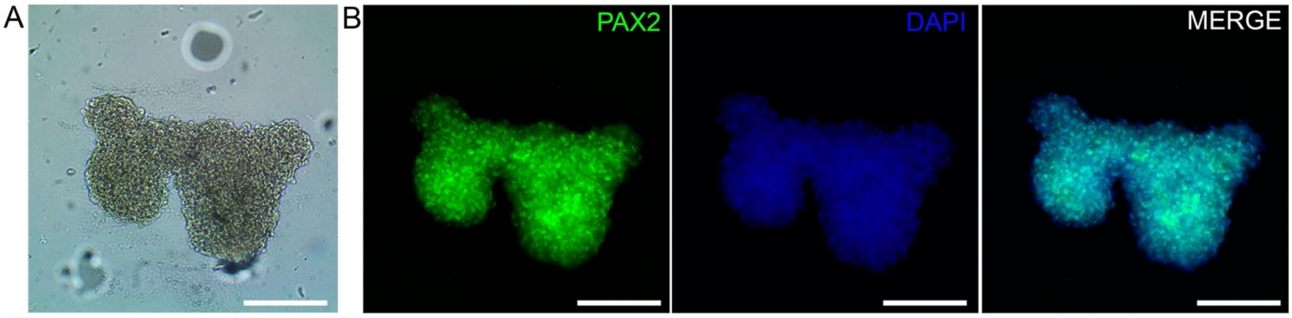

Supplementary Figure 4. Pax2 expression in ARPC spheroids. (A) Renal spheroids formed by ARPCs observed at phase contrast microscope. **(B)** Whole-mounting immunofluorescence showing nuclei stained with DAPI (blu) Pax2 (green) in ARPC spheroids. The scale bars represent 50 μm.

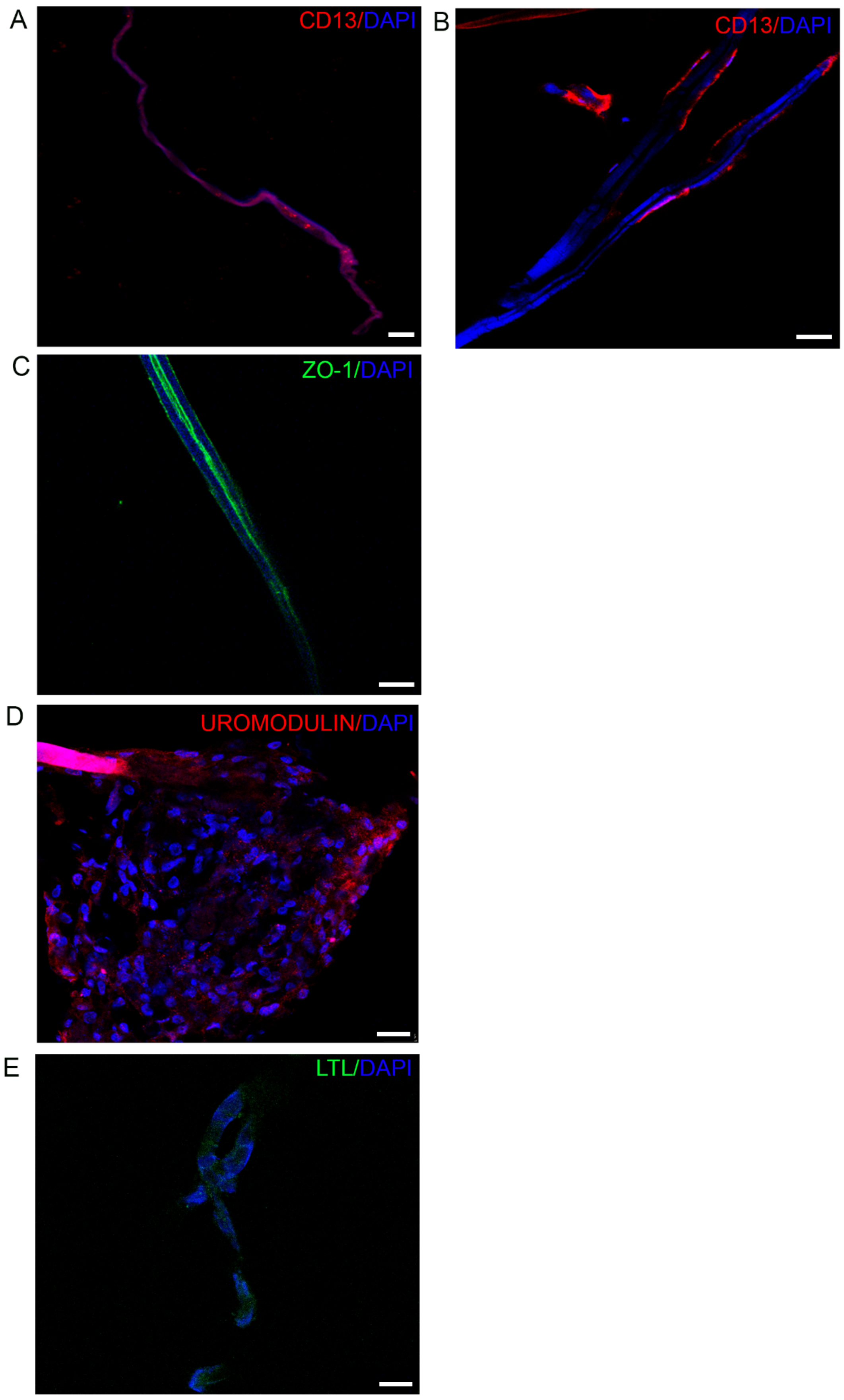

Supplemental Figure 5. Expression of renal tubular markers in tubular-like structures.

(A-B) Whole-mount immunofluorescence showing that the tubule-like structures generated from spheroids were positive for CD13 (aminopeptidase N). (C) Whole-mount immunofluorescence showing the expression of ZO-1 in tubular-like structures. (D) Whole-mount immunofluorescence showing the expression of uromodulin in a tubule-like structure that exits the spheroid. (E) Whole-mount immunofluorescence showing the expression of lotus tetragonolobus lectin (LTL) in tubular-like segments forming loop structures. The scale bars represent 25 μm in A-C, and 10 μm in D-E. Cell nuclei were visualized using DAPI (blue).

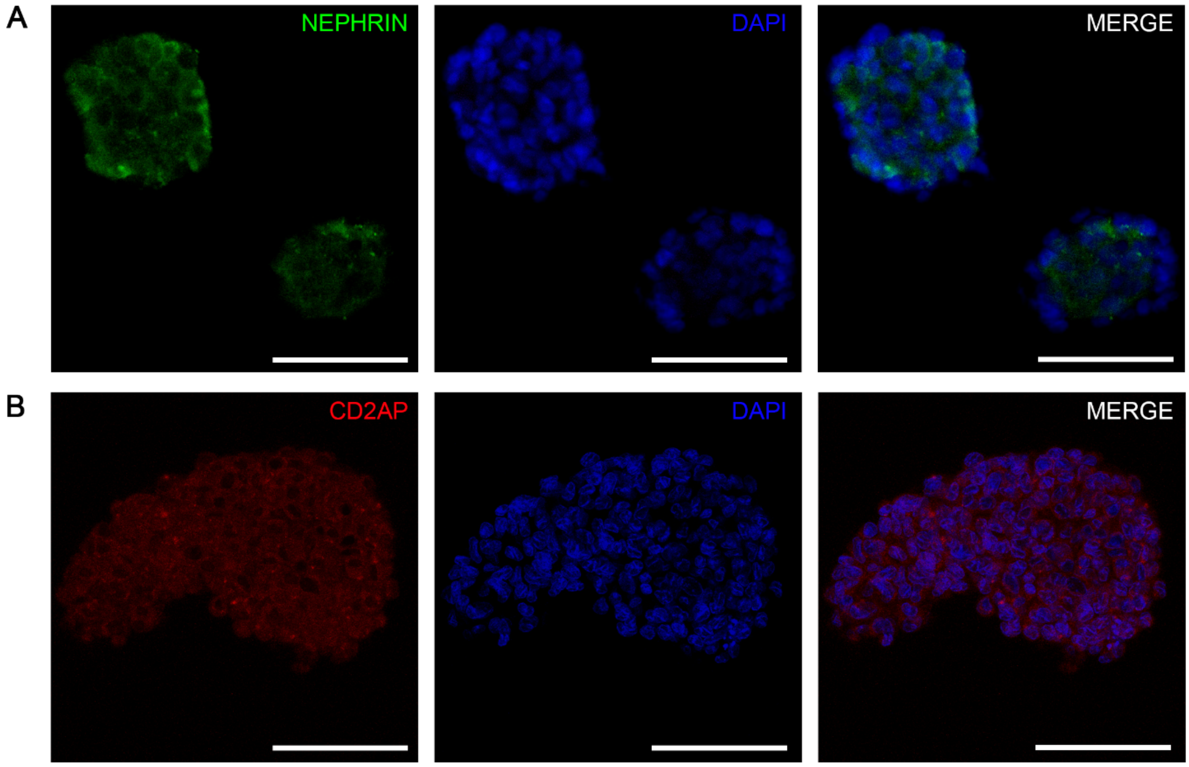

Supplemental Figure 6. Expression of glomerular markers in ARPC spheroids. (A) Whole-mount immunofluorescence showing the expression of nephrin in spheroids generated by ARPCs. **(B)** Whole-mount immunofluorescence showing the expression of CD2AP in spheroids generated by ARPCs. The scale bars represent 100 μm in A and 50 μm in B. Cell nuclei were visualized using DAPI (blue).
